# TRPV4 regulates osteoblast differentiation and mitochondrial function that are relevant for channelopathy

**DOI:** 10.3389/fcell.2023.1066788

**Published:** 2023-06-12

**Authors:** Tusar Kanta Acharya, Subhashis Pal, Arijit Ghosh, Shamit Kumar, Satish Kumar, Naibedya Chattopadhyay, Chandan Goswami

**Affiliations:** ^1^ National Institute of Science Education and Research, HBNI, School of Biological Sciences, Bhubaneswar, Odisha, India; ^2^ Training School Complex, Homi Bhabha National Institute, Mumbai, India; ^3^ Division of Endocrinology and Center for Research in Anabolic Skeletal Target in Health and Illness (ASTHI), Central Drug Research Institute (CDRI), Council of Scientific and Industrial Research (CSIR), Lucknow, India; ^4^ AcSIR, CSIR-Central Drug Research Institute Campus, Lucknow, India

**Keywords:** mitochondria, channelopathy, bone, genetic disorder, bio-mineralization

## Abstract

Different ion channels present in the osteoblast regulate the cellular functions including bio-mineralization, a process that is a highly stochastic event. Cellular events and molecular signaling involved in such process is poorly understood. Here we demonstrate that TRPV4, a mechanosensitive ion channel is endogenously present in an osteoblast cell line (MC3T3-E1) and in primary osteoblasts. Pharmacological activation of TRPV4 enhanced intracellular Ca^2+^-level, expression of osteoblast-specific genes and caused increased bio-mineralization. TRPV4 activation also affects mitochondrial Ca^2+^-levels and mitochondrial metabolisms. We further demonstrate that different point mutants of TRPV4 induce different mitochondrial morphology and have different levels of mitochondrial translocation, collectively suggesting that TRPV4-mutation-induced bone disorders and other channelopathies are mostly due to mitochondrial abnormalities. These findings may have broad biomedical implications.

## Introduction

The physiological balance between osteoblasts and osteoclasts, both in numbers as well as in activities is critical for maintenance of the skeletal integrity and also for remodelling. While osteoclasts are bone-resorbing cells, osteoblasts strengthen the skeletal system by enhancing the deposition of Ca^2+^-complexes (Matsuo and Irie, 2008). Derived from Mesenchymal Stem Cells (MSCs), osteoblasts remodel the skeletal system via new bone formation by deposition of several yet predominantly Ca^2+^-ions that promote biomineralization. In this context, several hormones play diverse roles in bone health and is also a direct reflection of endocrinological status (Rizzoli and Bonjour, 1997). The imbalance between osteoblast-osteoclast crosstalk causes skeletal deformities of several kinds, like osteoporosis or osteopetrosis (Zaidi, 2007). It becomes crucial to maintain the physiological homeostasis of the bone modelling cells. Different Ca^2+^ channels, like voltage-gated Ca^2+^-channels (VGCCs), L-type Ca^2+^ channels, ryanodine receptors (RyR), or inositol-1,4,5-trisphosphate receptors (IP_3_R) also play important roles in case of osteoblast functions (Robinson et al., 2010).

Transient Receptor Potential Vanilloid subtype 4 (TRPV4) is a member of TRP ion channel superfamily. Though it acts as a “non-selective” cation channel and is permeable to different mono- and divalent cations albeit with higher permeability for Ca^2+^ ions that enables it to induce various Ca^2+^-signaling events. TRPV4 is also “mechanically” activated, making it a unique candidate for sensing mechanical force related to bone cells (Guilak et al., 2010). TRPV4 is also activated by temperature in the physiological range. Notably, TRPV4 represents a very ancient ion channel present in all vertebrates and its protein sequence is highly conserved. Most invertebrates also have functional homologs that are critically different from vertebrate-specific TRPV4. In human, the TRPV4 gene is located at chromosome 12 and this synteny is a highly disease-prone region (Kumari et al., 2015). Notably, the TRPV4-gene containing syntenic organization is also highly conserved in all vertebrates, suggesting that the genes present in this locus as well as TRPV4 play a critical role in all vertebrates. The TRPV4 gene containing synteny has several other important genes, many of these are highly linked with different diseases. Also the same synteny has at least two genes (MVK and GLTP) that are involved in cholesterol and lipid metabolism. Notably, TRPV4 function is critically regulated by cholesterol and other lipids. Accordingly, naturally occurring point mutations of TRPV4 (also affecting the cholesterol interaction) lead to bone and muscle defects (Das and Goswami, 2019; Das and Goswami, 2022; Das et al., 2023).

TRPV4 interacts with cholesterol, which is also important for bone formation (Kumari et al., 2015; Rendina-Ruedy and Rosen, 2020). Indeed, cholesterol-mediated regulation of TRPV4 is important for TRPV4 functions and mechanosensitivity regulation (Lakk et al., 2021). Not only cholesterol, TRPV4 also interacts with the metabolic intermediates of cholesterol, such as mevalonate and derivatives such as steroids and Cytochrome C, a mitochondrial protein through its TM4-loop-TM5 region which is highly conserved in all vertebrates (Kumari et al., 2015; Das et al., 2022). Notably, a large number of point mutations have been identified in this region (TM4-loop-TM5) of TRPV4, which causes a series of musculoskeletal disorders (Nilius and Voets, 2013; Leddy et al., 2014). Notably, TRPV4-R616Q (pathogenic and a gain-of-function type mutant of TRPV4) has less interaction with cholesterol, suggesting that loss-of-interaction can cause more channel opening (Das and Goswami, 2019). In the majority of these cases, the mutation affects bone development and functions, strongly suggesting that TRPV4 plays a role in osteoblasts. However, the cellular mechanisms are not understood yet.

Using TRPV4 knock out animals (*Trpv4* ^*−/−*^), importance of TRPV4 in the maintenance of bone mass has been reported although the findings lack consensus (Suzuki et al., 2013; Nishimura et al., 2020). Moreover, mice deficient in both *Trpv1* ^*−/−*^ and *Trpv4* ^*−/−*^ have increased osteoblast differentiation and bone mass (Nishimura et al., 2020). Considering that TRPV4 is present in several tissues (including kidney and urinary balder which are critical in maintaining body’s Ca^2+^ homeostasis) and cells (such as adipocytes, macrophages, T cells, chondrocytes, etc.), the exact role of TRPV4 in bone mineralization is difficult to ascertain, especially using knock out animals (Du et al., 2020; Acharya et al., 2022c). Therefore, in this study, we explored the importance of TRPV4 in bone mineralization using osteoblast cell lines as well as primary cultures where TRPV4 is endogenously expressed. These cell culture systems are arguably suitable to address the role of TRPV4 in bone mineralization without the interference of non-osteoblastic ells such as immune cells and adipocytes that affect the bone formation process *in vivo*. In this study, we explored the endogenous expression and function of TRPV4 in osteoblasts. We further explored if TRPV4 activation alters subcellular organelle, such as mitochondrial functions. Lastly, we characterized different pathogenic mutants of TRPV4 in the context of mitochondrial morphology and function.

## Materials and methods

### Cell line and primary cell culture

MC3T3-E1 cells (sub-clone 14) were maintained in α-MEM media (Sigma Aldrich) supplemented with 10% Fetal Bovine Serum (FBS, HiMedia) and 100 units/mL Penicillin + 100 μg/mL Streptomycin solution (HiMedia). For preparing differentiation media, 10 mM β-glycerophosphate, 10 nM Dexamethasone and 100 μg/ml L-ascorbic acid (purchased from Sigma Aldrich) were added to the above-mentioned media.

Sprague-Dawley (SD) rat pups (0–2 days old) were procured from NISER animal house facility and sacrificed by cervical dislocation. Calvaria was carefully taken out, washed with PBS, muscles and blood were removed. The calvarial fragments were then subjected to enzymatic digestion by incubating in 10 mg/mL collagenase + dispase solution (purchased from Sigma) in 37°C water bath for 1.5 h with gentle shaking. Cells were collected after passing the enzyme digested tissue suspension through a 70 μm cell strainer. Cells were cultured in α-MEM supplemented with 10% FBS. For the isolation of mouse bone marrow-derived mesenchymal stem cells (MSC), we have followed the protocol as described before (Acharya et al., 2021).

Transient transfection was performed by Lipofectamine 3,000 Plus reagent (Invitrogen) according to the manufacturer’s protocol. Typically, 24–36 h after transfection, the cells were used for live-cell imaging or immunocytochemistry.

### Alkaline phosphatase assay

Alkaline Phosphatase Assay (ALP assay) media was prepared by adding 100 μg/ml L-ascorbic acid and 10 mM β-glycerophosphate to normal culture media. The ALP assay was performed by seeding equal number of cells (RCOs and MC3T3-E1) in 96-well plates (Corning) and culturing in ALP-media for 2 days in presence or absence of drugs. The RCOs were treated with different concentrations of 4αPDD (10 pM, 100 pM, 1 nM, 10 nM, 100 nM, 1 µM) and RN1734 (10 pM, 100 pM, 1 nM, 10 nM, 100 nM, 1 µM) along with ALP media. Similarly, the MC3T3-E1 cells were treated with (0.1 nM, 1 nM, 10 nM, 100 nM, 1 µM) and RN1734 (0.1 nM, 1 nM, 10 nM, 100 nM, 1 µM) with ALP media. After 2 days, media was aspirated, and cells were washed with PBS. Cells were lysed by subjecting the plate to repeated freeze-thaw cycle and ALP substrate (purchased from Sigma Aldrich, cat#N2770) was put in equal quantity in each plate. After 2–3 h of incubation, dark color develops. Subsequently the spectrophotometric reading was taken in 405 nm in an ELISA plate reader (VarioSkan, Thermo scientific).

### Mineralization assay

Mineralization media was prepared by adding 10 mM β-glycerophosphate, 100 μg/ml L-ascorbic acid and 10 nM Dexamethasone (Sigma) to normal culture media (Siddiqui et al., 2010; Siddiqui et al., 2011). Mineralization assay was performed by seeding 1 × 10^5^ number of cells (RCOs) in 12-well culture plates and cultured in mineralization media for 21 days in the presence of TRPV4 channel agonist 4αPDD (10 nM). After 21 days, media was aspirated, cells were washed with PBS and an equal amount of Alizarin Red-S (ARS) dye (Sigma Aldrich, cat#A5533) was added to each well. After 30 min of incubation with ARS, the dye was washed away with water and micrographs were taken to visualise the extent of mineralization in different conditions as required in individual experiments. For quantifying the extent of mineralization, Cetyl Peridynium Chloride (CPC, purchased from MP Biomedicals) were added in each well. The dye was extracted in CPC and spectrophotometric reading was taken at 550 nm. In another condition mouse bone marrow derived MSCs were treated with 4αPDD (1 µM) only and RN1734 (1 µM) + 4αPDD (1 µM) for 15 days along with osteogenic differentiation media. In a similar manner, for mineralization of MSCs, we used GSK1016790A (10 nM, 100 nM, 1 µM) as another specific agonist of TRPV4. ARS staining and CPC extraction and quantification was performed as mentioned above.

### Immunocytochemistry

Cells were cultured on 12 mm cover slips in 24 well plates. After differentiation, cells were fixed with 4% Paraformaldehyde (PFA, purchased from Sigma) for 15 min in room temperature (∼25°C). PFA was then aspirated from the wells and cells were washed three times with PBS. Cells were then permeabilized with 0.1% Triton X-100 in PBS (1X) for 4 min and blocked with 5% Bovine Serum Albumin (BSA) for 30 min. The coverslips were then washed thrice with 200 μL 0.1% PBS-T for 5 min each. After washing with PBS-T, cells were probed with the anti-TRPV4 antibodies (Alomone Labs, Israel, Cat#ACC-034) at a dilution of 1:250 in different experimental settings. Alexa Fluor-488 conjugated secondary antibody (Invitrogen) with 1:500 dilution was used to visualize TRPV4 channel and DAPI (5 μM) was used as a counterstain to visualize the nucleus. For confirming the specificity of the antibodies for the TRPV4 channel, epitope-specific peptide procured from Alomone Labs (Cat#BLP-CC034) were used. Firstly, the antibodies were incubated with respective antigenic peptides for 30 min and then cells were probed with the antigen-antibody complex formation.

### Live cell Ca^2+^-imaging

For live cell imaging of basal Ca^2+^-level, rat calvarial osteoblasts (RCOs) were plated on 25 mm coverslips in 35 mm tissue culture dishes (Corning) and cultured in differentiation media for 3 days. Cells were treated with pharmacological activator of TRPV4 (4αPDD, 10 nM) for 2 days. Subsequently, cells were treated with 0.02% Pluronic^®^ F-127 (Thermo Fisher Scientific) and 5 μM Fluo-4 AM (Molecular Probe) for 45 min and live-cell imaging was then performed in a Zeiss LSM 780 confocal microscope.

For the measurement of cytosolic Ca^2+^ level the MC3T3-E1 cells were transfected with genetically encoded Ca^2+^-sensor GCaMP. Post 24 h. of transfection, the Ca^2+^-imaging was performed by using 4αPDD (5 µM) and RN1734 (10 µM). The drugs were added on the 20th frame of the time series and the whole live cell imaging was recorded up to 200th frame (∼3.5 min). We further treated the cells with RN1734 (10 µM) for 1 h and subsequently the cells were activated by 4αPDD (5 µM) (without washing RN1734) and the Ca^2+^-influx was recorded up to 200th frame (equivalent to ∼3.5 min). Higher concentration of drugs was used to check the instant activation of TRPV4 channel. The change in calcium intensity was quantified by ImageJ.

For measurement of cytosolic Ca^2+^ in MSCs, the cells were labelled with Fluo-4-AM dye (5 μM) and live cell Ca^2+^ imaging was performed by adding GSK1016790A (1 µM) at the 20th frame. In corresponding control experiments, the cells were pre-incubated with RN1734 (10 μM for 1 h) and instant Ca^2+^ influx was measured using GSK1016790A (without washing RN1734) as the stimulus.

### qRT-PCR analysis

Quantitative real-time polymerase chain reaction (qPCR) was performed to determine the relative expressions of osteoblast specific genes in drug-treated RCOs. RCOs were treated with 4αPDD (10 nM) for 48 h along with α-MEM media (Sigma Aldrich) supplemented with 10% FBS and 100 units/mL Penicillin + 100 μg/mL Streptomycin solution. GAPDH was used as the internal control. Primers were designed by the Universal Probe Library (Roche Applied Science) for the following genes: RunX2, Bmp2, and Col1. The primer list is described previously (Acharya et al., 2021). All cDNAs were synthesized with Revert Aid cDNA synthesis kit (Fermentas, Austin, United States) using 2 µg total RNA. SYBR green chemistry was employed to perform quantitative detection of relative expression of mRNA levels of these genes using a Light Cycler 480 (Roche Molecular Biochemicals, Indianapolis, United States) as described before (Siddiqui et al., 2010).

### SDS-PAGE and western blot analysis

For the preparation of gel sample, the RCOs grown in differentiation media for 7 days were harvested. The gel sample is prepared by adding Laemmli buffer supplemented with protease inhibitor cocktail (Sigma) and subsequently samples were boiled for 5 min at 95°C. The samples were analyzed by 10% SDS-PAGE and followed by Western blot analysis as described before (Kumar et al., 2016; Acharya et al., 2021).

### Glycolysis and mitochondrial respiration

Glycolysis and mitochondrial respiration were quantified using a previously described procedure (Porwal et al., 2017). Briefly, rat calvarial osteoblast were cultured in α-MEM with 10% FBS. Glycolysis and mitochondrial respiration experiment was carried out without the presence of mineralization media. 48 h prior to assay 40,000 cells/well were plated in a 24-well polystyrene Seahorse V7-PS Flux plate with no additional coating and treated with 4αPDD (10 nM). Before starting the assay, cells were washed twice with assay media (XF Base minimal DMEM, catalog number 98546008) and kept at 37°C for 1 h. Glycolysis was analyzed by stepwise addition of A: glucose (10 mM), B: oligomycin (1 μM) and C: 2-deoxy-D-glucose (50 mM). Mitochondrial respiration was assayed by stepwise addition of A: oligomycin (1 μM), B: carbonyl cyanide-4-(trifluoromethoxy) phenylhydrazone (FCCP) (2 μM) and C: rotenone (0.5 μM). Three measurement cycles of 2 min mix, 1 min wait, and 5 min measure were carried out after each addition.

### Mitochondrial Ca^2+^-imaging

Mitochondrial Ca^2+^-imaging was performed with ratiometric Mito-Pericam constructs (A gift from Dr. Atsushi Miyawaki, Saitama, Japan). Ratiometric Mito-Pericam is a Ca^2+^-indicator made by fusion of the yellow fluorescent protein (YFP) and Calmodulin which is able to translocate within mitochondria (Nagai et al., 2001). The binding of Ca^2+^ to Mito-Pericam changes its excitation wavelengths from 415 nm to 494 nm, while its emission spectrum is maintained at 515 nm. Mito-Pericam was expressed in MC3T3-E1 cells by transient transfection and 24 h after transfection. Cells were imaged with a confocal microscope. Ca^2+^-imaging was performed by adding 4αPDD (5 µM), RN1734 (10 µM) at the 20th frame during the imaging. In some experiments, cells were pre-incubated with RN1734 (10 µM) for 1 h and subsequently activated by 4αPDD (5 µM). TRPV4 activator (4αPDD) was used at 5 μM (slightly higher than the optimum concentration) to assess the instantaneous influx of Ca^2+^ inside the mitochondria. Using particle-based quantification by ImageJ, we have measured the mitochondrial Ca^2+^- level. For that purpose, time-series imaging was performed (total of 200 frames (∼7.5 min) and 4αPDD was added on the 20th frame in each condition). Images at different time points (Frame-1, 20, 30, 50, 100, 150, and 200) were used to analyse the Ca^2+^-level. More than 18,000 mitochondrial particles from multiple cells were quantified in each frame.

### Mitochondrial morphology parameters estimation

For the quantification of mitochondrial morphological parameters, we used mitochondria morphology plugin (https://imagejdocu.tudor.lu/plugin/morphology/mitochondrial_morphology_macro_plug-in/start) as described before (Dagda et al., 2009). The quantification of ratio of mitoDsRed intensity with GFP intensity (of TRPV4-WT and TRPV4 mutants), fluorescence intensity for GFP as well as DsRed from individual mitochondria were analysed by ImageJ.

### Statistics

Statistical analysis were performed by using GraphPad Prism 7 (Version 7.00). Data represented in the graphs as mean ± SEM. One-way ANOVA test with Brown-Forsythe test, Bartlett’s test and Sidak’s multiple comparisons test, and Student’s *t*-test were done as and where required.

### Animal procurement and primary cell isolation

All the animal experiments were performed at the animal house facility of NISER and CDRI as per the guidelines of IAEC (NISER/SBS/AH/IAEC-56 & NISER/SBS/AH/IAEC-55, CDRI/IAEC/2013/34). All the experimental methods were carried out by following relevant guidelines and regulation of CPCSEA.

## Results

### TRPV4 is functionally expressed in primary rat calvarial osteoblasts and mouse osteoblast cell line

**FIGURE 1:**
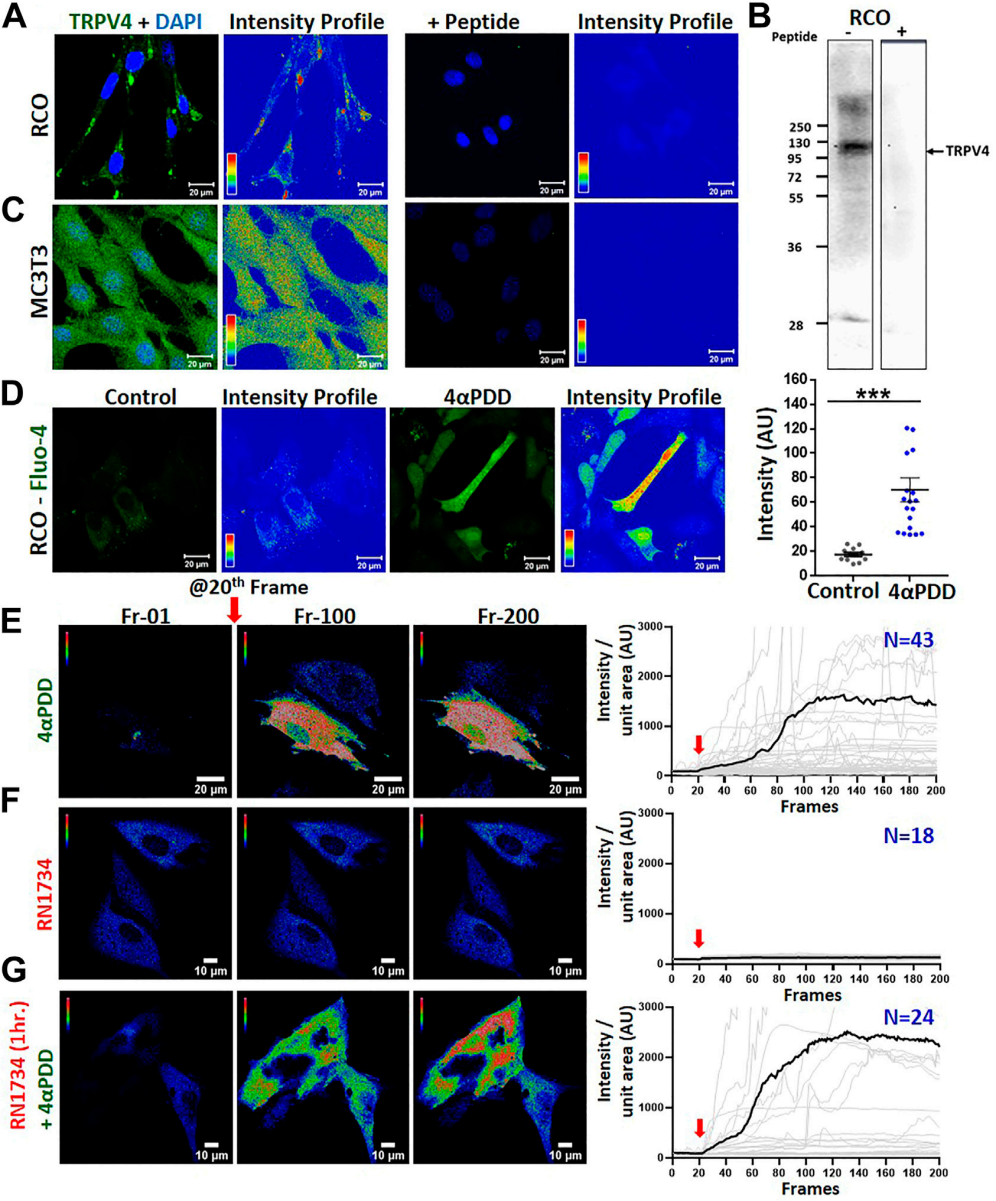
Activation of endogenous TRPV4 in osteoblasts results in instantaneous Ca^2+^-influx **(A–C)**. Shown are the immunofluorescence **(A,C)** and Western blot **(B)** analysis of RCO **(A,B)** & MC3T3 **(C)** in the absence and presence of a blocking peptide. **(D)**. Comparision of basal Ca^2+^-levels in RCOs in control and 4αPDD-activated condition. Fluorescence images and intensity profile are shown. The values were compared using Student’s *t*-test, ***p < 0.001 **(E,F)**. Instant Ca^2+^-influx was measured in MC3T3-E1 cells expressing Ca^2+^-sensor GCaMP by treating with 4αPDD or RN1734. Shown are intensity profile of frame 1, 100, and 200 of live cell Ca^2+^ imaging along with quantitative analysis. Addition of TRPV4 activator causes sudden increase in the cytosolic Ca^2+^-level. Quantitative representation of Ca^2+^-imaging. TRPV4 activation by 4αPDD **(E)** causes a rapid increase in Ca^2+^ level (N = 43) whereas, RN1734 addition **(F)** shows no change in Ca^2+^ level (N = 18) **(G)**. Cells when pre-incubated with RN1734 for 1 h and subsequently activated by 4αPDD, show more Ca^2+^-influx (N = 24).

We studied the endogenous expression of TRPV4 in rat calvarial osteoblasts (RCOs) by immunofluorescence and western blotting for which the specificity of primary antibodies has been confirmed by respective peptide block (Figures 1A, B). Similar immunofluorescence approach was applied for studying TRPV4 expression in murine pre-osteoblast cell line MC3T3-E1 (Figure 1C). Next, we studied the functionality of TRPV4 and thus performed Ca^2+^-imaging in MC3T3-E1 osteoblasts using a TRPV4 activator, 4αPDD. MC3T3-E1 cells were transiently transfected with GCaMP. 24 h after transfection, cells were treated with 4αPDD and we observed Ca^2+^-rise that sustained in a manner (Figure 1E). In a similar experiment, when cells were treated with a TRPV4 inhibitor, RN1734 (10 µM) the intracellular Ca^2+^-level was reduced (Figure 1F). However, when cells were pre-treated with RN1734 (10 µM) for 1 h and subsequently treated with 4αPDD, the intracellular Ca^2+^-level was further elevated (Figure 1G). In differentiating RCOs, the basal cytosolic Ca^2+^-levels increased in the 4αPDD (10 nM) treated cells (Figure 1D). Instantaneous Ca^2+^-influx in response to GSK1016790A (1 µM, as an another agonist for TRPV4) in MSC was also observed (Supplementary Figure S1). When cells were pre-treated with RN1734 (10 µM, for 1 h), Ca^2+^ influx in response to GSK1016790A was also observed, mainly due to lack of Ca^2+^-mediated desensitization (discussed later). We observed elevated Ca^2+^ level in both the experimental conditions, but the presence of RN1734 dampened the Ca^2+^-influx faster as compared to only GSK1016790A (Supplementary Figure S1).

### Pharmacological modulation of TRPV4 increases osteoblast differentiation

**FIGURE 2:**
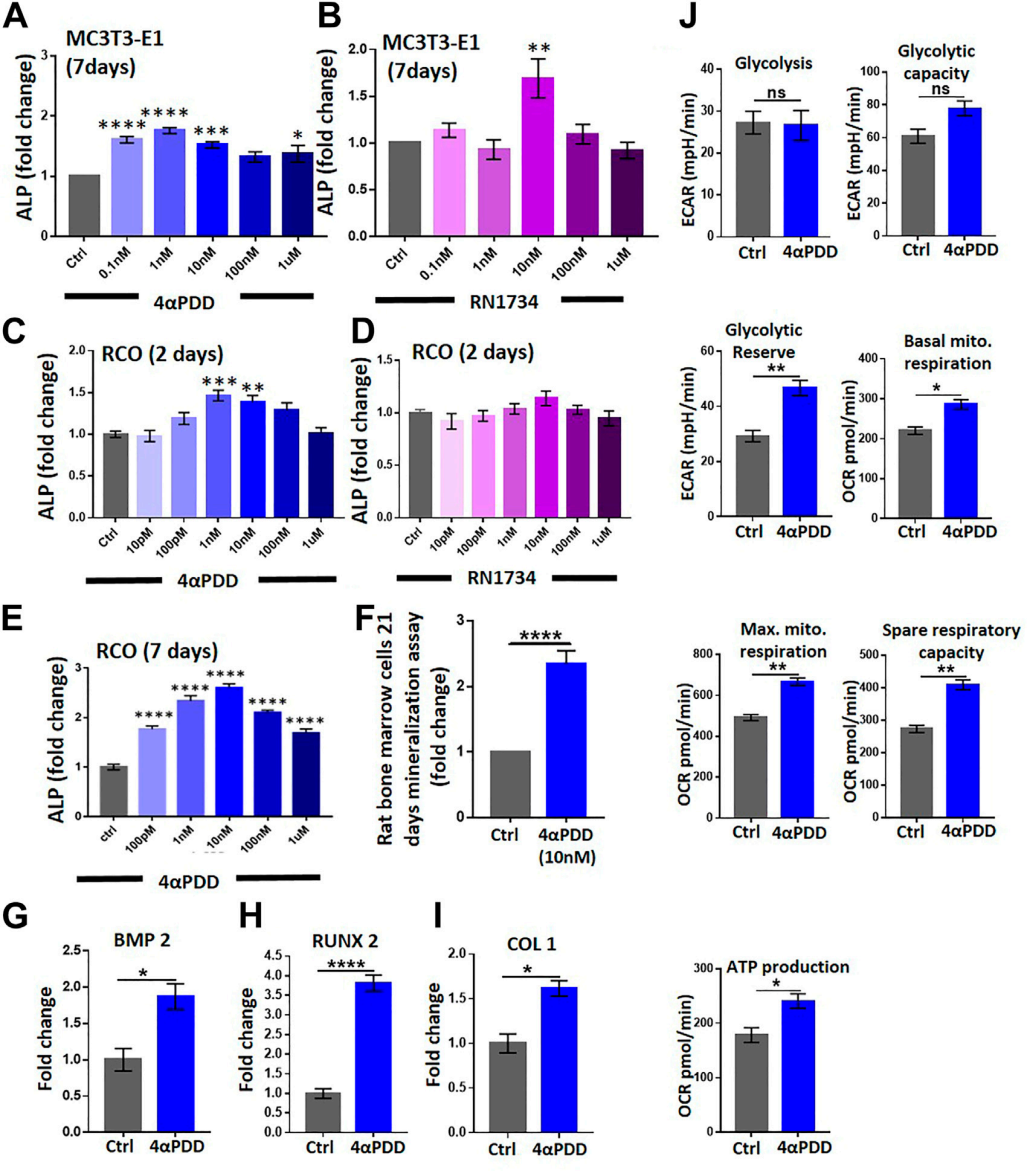
TRPV4 activation promotes osteogenic differentiation and affects mitochondrial energetic and respiration. **(A)**. ALP analysis of MC3T3-E1 cells (7 Days) in presence of increasing concentration of TRPV4 activator 4αPDD **(B)**. ALP analysis by using TRPV4 inhibitor RN1734 in MC3T3-E1 cells **(C)**. ALP analysis of RCO (2 Days) in presence of increasing concentration of TRPV4 activator 4αPDD **(D)**. ALP assay was carried out by using TRPV4 inhibitor RN1734 **(E)**. ALP analysis of RCO (7 Days) in presence of increasing concentration of TRPV4 activator 4αPDD. (* = p < 0.05, ** = p < 0.01, *** = p < 0.001, **** = p < 0.0001, one-way ANOVA) **(F)**. TRPV4 activation leads to increased nodule formation as assessed by Alizarin Red-S assay **(G–I)**. TRPV4 activation leads to increased expression of osteogenic genes (BMP2, RUNX2, COL1) **(J)**. TRPV4 activation leads to increased mitochondrial parameters. Average values of biological repeats (*n* = 3) are shown. The values are Student’s *t*-test, ns: non-significant, * = p< 0.05, ** = p< 0.01, **** = p < 0.0001.

We investigated the role of TRPV4 in the differentiation in MC3T3-E1 cells. To this aim, we performed an alkaline phosphatase (ALP, 7 days) assay, a marker of osteoblast differentiation. MC3T3-E1 cells were cultured with or without 4αPDD or RN1734 in increasing concentrations (Figures 2A, B). However, the increment of ALP intensity remains unpredictable, especially in certain doses of RN1734 conditions. This might be due to complex biological variation and/or some unexplained reasons. We observed that 4αPDD increased the differentiation of MC3T3-E1 cells by ∼1.7 folds compared with DMSO control (Figure 2A). We next studied if TRPV4 modulated the differentiation of RCOs. To this aim, RCOs were cultured in the presence of increasing concentration of 4αPDD or RN1734 (10 pM to 1 μM) for 2 days and then cells were fixed in 4% PFA (Figures 2C, D). TRPV4 activation significantly promoted differentiation of RCOs (at 1 nM, 4αPDD increased differentiation by ∼1.4 folds) compared with DMSO control (Figure 2C). Moreover, 4αPDD (10 nM) increased the differentiation of RCOs at day 7 by ∼2.7 folds compared with DMSO control (Figure 2E).

### TRPV4 activation results in increased mineralization by rat bone marrow stromal cells

We next studied the ability of 4αPDD (10 nM) in stimulating the formation of *ex vivo* mineralized nodule formation by bone marrow stromal cells obtained from rats. As shown in (Figure 2F), 4αPDD increased mineralized nodule formation by ∼2.5-fold compared with control cells. In the same condition, cells were activated with 4αPDD alone (at 1 μM) or in presence of RN1734 (1 μM). We noted that 4αPDD alone causes more mineralization as compared to the control. However, presence of RN1734 reduces the efficacy of 4αPDD-mediated mineralization significantly (∼42%). Therefore, RN1734 can reverse the activity of 4αPDD (Supplementary Figure S2). Using a similar protocol, we have performed induction of *in vitro* mineralization in MSC using another TRPV4 agonist GSK1016790A (10 nM, 100 nM, 1 µM). GSK1016790A treatment increased mineralized nodules as compared to control condition (Supplementary Figures S3A, B).

Moreover, in RCOs, 4αPDD (10 nM, 48 h) significantly increased the mRNA levels of osteogenic genes including runt-related transcription factor 2 (*Runx2*), bone Morphogenic protein-2 (*Bmp2*), and collagen type-1 (*Col1*) (Figures 2G–I). Taken together, data suggests that TRPV4 activation induces the expression of osteogenic genes required for osteogenic differentiation.

### TRPV4 modulation alters osteoblast energetics

Osteoblast differentiation is a highly energy-intensive process and it uses both glycolysis and mitochondrial respiration to fuel the process (Guntur et al., 2014). Therefore, we studied the effect of TRPV4 activation on the regulation of energetics of RCO. Using extracellular flux analyzer, we observed that TRPV4 activation increased basal glycolysis (extracellular acidification), glycolytic reserve and capacity, under normal glucose concentration (1 mg/mL) (Figure 2J). Next, we studied the effects of TRPV4 activation in mitochondrial respiration. TRPV4 activation increased both basal and maximum mitochondrial respiration (oxygen consumption), spare respiratory capacity and ATP production in osteoblasts (Figure 2J).

### “Gain-of-function” mutants of TRPV4 affect mitochondria differently

**FIGURE 3:**
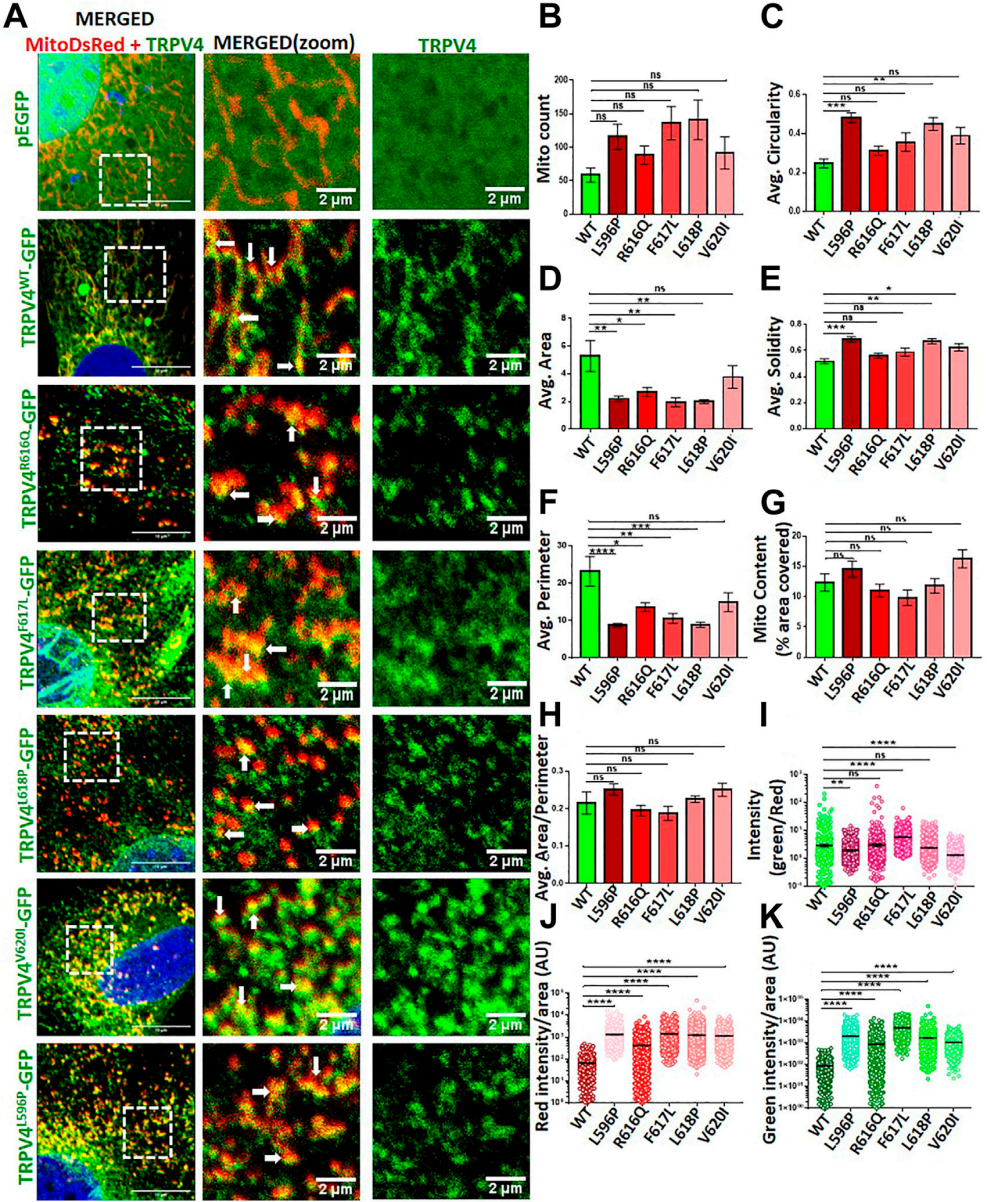
TRPV4 mutants differentially affect mitochondrial function **(A)**. Shown are the confocal images of an enlarged area of MC3T3-E1 cells expressing mitoDsRed (red)/TRPV4-WT-GFP/different mutants/only GFP (green). Areas of co-localization of TRPV4 with mitoDsRed are indicated by arrows **(B–H)**. Different parameters of mitochondria in cells expressing TRPV4-WT or different mutants were compared **(I)**. TRPV4 enrichment in mitochondria was analyzed by quantifying the ratio of TRPV4-GFP intensity over mitoDsRed in each mitochondrion (*n* = ∼1,000 individual mitochondria from ∼10 cells). The values were compared as one-way ANOVA, ns: non-significant, * = p< 0.05, ** = p< 0.01, *** = p< 0.001, **** = p< 0.0001 **(J,K)**. Import of mitoDsRed or TRPV4 were analyzed by quantifying the intensity of TRPV4-GFP-WT or different mutants and mitoDsRed in each mitochondrion (*n* = ∼1,000 individual mitochondria from ∼10 cells). The values are one-way ANOVA, ns: non-significant, ** = p< 0.01, *** = <0.001, **** = <0.0001.

Several point mutations in TRPV4 are known to induce musculoskeletal defects (Verma et al., 2010). In order to assess for the impact of TRPV4 mutations on the mitochondrial function, we co-expressed full-length wild-type (WT) or a series of TRPV4 point mutants (R616Q, F617L, L618P, V620I, L596P) along with a marker for mitochondria (i.e., mitoDsRed) in MC3T3-E1 cells by transient transfection (Figure 3A). In all these cases, colocalization of TRPV4 with mitoDsRed was observed albeit with variable extent. We then quantified the parameters including mitochondrial number, size, distribution, roundness and protein import that determine mitochondrial health. Mitochondrial number and distribution in osteoblasts did not alter with the altered TRPV4 function (Figure 3B). However, both area and perimeter of the mitochondria were reduced in the presence of most of the TRPV4 mutants (Figures 3D, F). The mito content and the overall area-perimeter ratio were also calculated (Figures 3G,H). Both solidity and circularity of mitochondria, indicative of their morphology, were affected in the presence of specific mutants. L596P, L618P, and V620I mutations increased the solidity of mitochondria (Figures 3C, E), whereas L596P and L618P mutations increased mitochondrial circularity (Figure 3C). To understand the mitochondrial translocation/import, we measured the intensity of TRPV4 as well as mitoDsRed for cells expressing TRPV4-WT or different mutants. Both mitoDsRed and TRPV4 fluorescence intensities were increased in the presence of mutations (Figures 3I–K). The ratio of TRPV4-GFP intensity with mitoDsRed intensity, however, was increased only in the presence of F617L, while it was decreased in L596P and V620I mutations, suggesting that different mutants affect the mitochondrial import in different extent (Figures 3I–K).

### TRPV4 activation affects the mitochondrial Ca^2+^-dynamics

**FIGURE 4:**
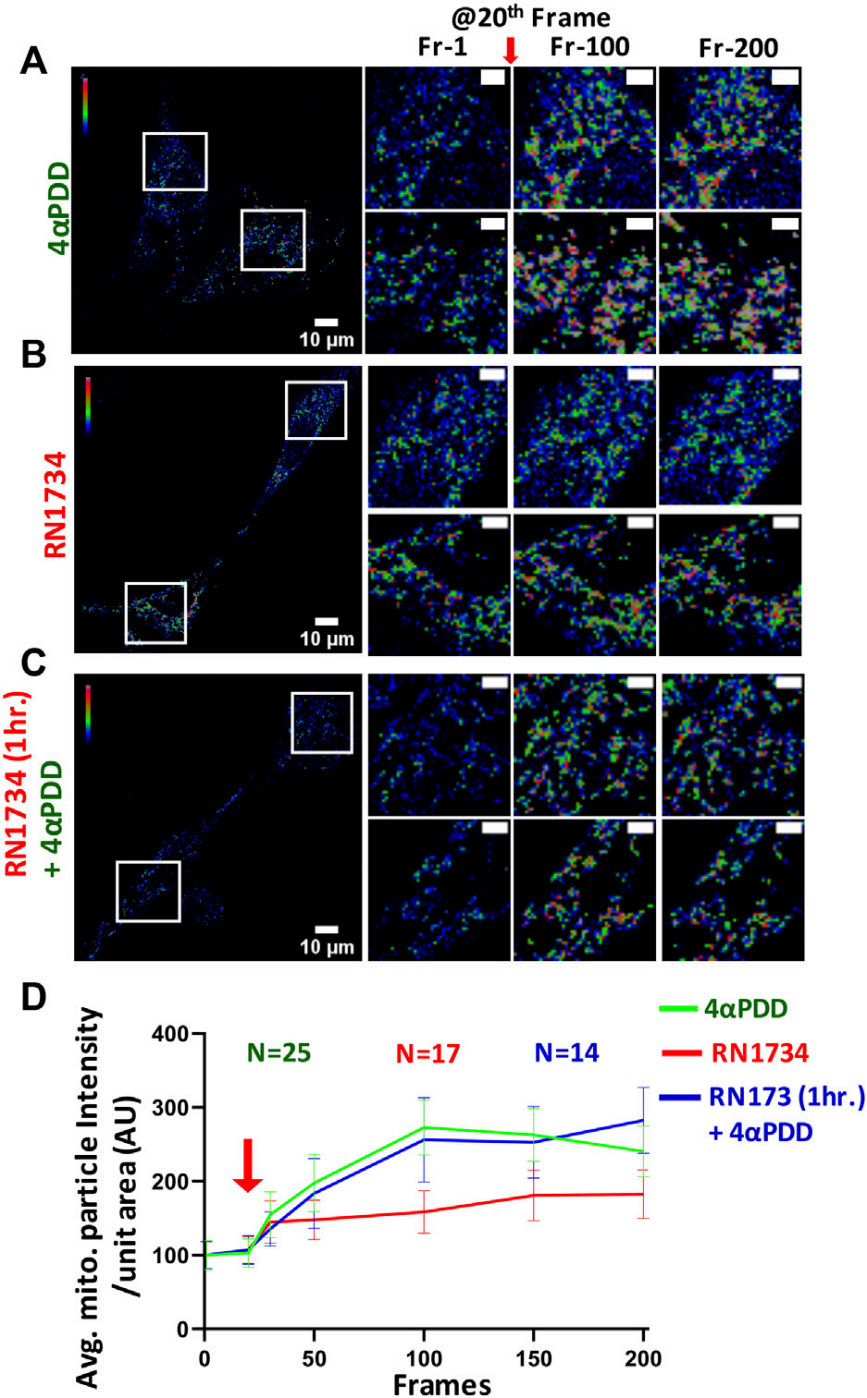
Activation of TRPV4 increases mitochondrial Ca^2+^-instantantly **(A–C)**. Shown are instant change in fluorescence intensity of MCT3T-E1 cells expressing MitoPeriCam, a mitochondrial Ca^2+^-sensor. Enlarged areas ar shown in the right side. TRPV4-activation by 4αPDD causes immediate increase in the mitochondrial Ca^2+^-levels. Addition of RN1734 leads to a slight increase in mitochondrial Ca^2+^-levels. Cells that were pre-incubated with RN1734 for 1 h and subsequently activated by 4αPDD also show surge in mitochondrial Ca^2+^. Mitochondrial particle intensity in different frames were quantified by ImageJ (≥18,000 total mitochondrial particles from multiple cells in each frame were analyzed). Scale bar 10 μm and 4 µm (for enlarged images) **(D)**. Quantitative representation of mitochondrial Ca2+-imaging is shown. Values at time frame: 1, 20, 30, 50, 100, 150, and 200 are quantified. Red arrow indicates the time point (20th frame) when TRPV4 activator is added.

Mitochondrial Ca^2+^-level is a critical parameter for the function of osteoblasts. Thus, we characterized the level of mitochondrial Ca^2+^ upon pharmacological activation of TRPV4. Our data show that mitochondrial Ca^2+^ was significantly increased upon the activation of TRPV4 by 4αPDD (5 µM) in MC3T3-E1 cells (Figures 4A, D). By contrast, when cells were treated with RN1734 (10 µM), marginal Ca^2+^-influx was observed (Figures 4B, D). Further, we treated the cells with RN1734 (10 µM) for 1 h and subsequently treated with 4αPDD (5 µM). In this condition also, increase in the mitochondrial Ca^2+^ was observed suggesting that TRPV4 as a regulator of mitochondrial Ca^+2^-levels in osteoblasts (Figures 4C, D).

## Discussion

Pre-osteoblast differentiation into mature osteoblasts and subsequent mineralization are the results of intricate cell signalling events that are stochastic in nature (Wang et al., 2022). Both intracellular Ca^2+^-levels as well as intracellular Ca^2+^-oscillation are important for these processes (Lieben and Carmeliet, 2012). Besides, Ca^2+^ channels regulate mechanical force and hormonal signaling (McDonald, 2004; Jiao et al., 2019), however, the underlying molecular and cellular mechanisms are less understood (Wang et al., 2022). In this context, TRP ion channels represent potential candidates as these channels are not only Ca^2+^-permeable, but are also “the molecular sensors” for different physical stimuli such as temperature and mechanical forces (Liu and Montell, 2015; Acharya et al., 2021). In this study, we demonstrate that TRPV4 is endogenously present in primary osteoblasts and also in the osteoblast line. Activation of TRPV4 leads to increased osteoblastic differentiation, Ca^2+^-deposition, and the expression of key genes (Runx2, Bmp2, Col1) specific for bio-mineralization process.

Previous reports suggest that temperature and mechanical force affect the bone functions (Brookes and May, 1972). Similarly, regular exercise increases bone mass (Benedetti et al., 2018). Astronauts and other frequent flyers experiencing zero or less gravity suffer from bone loss (Genah et al., 2021). In this context, involvement of TRPV4 (and also other TRP channels) has been studied in micro-gravity-induced bone loss (Bryant et al., 2017; Franco-Obregón et al., 2018). TRPV4 is important as it acts as a mechanical pressure sensor and gets activated by physiological temperature, membrane tension, and mechanical force along with other chemical stimuli. The above-mentioned observations open up possibilities for utilizing TRPV4 (including other TRP channels) as potential molecular target/s for bone-loss condition such as osteoporosis (McNulty et al., 2015). Recently, it has been reported that during RCO differentiation, the expression and distribution of TRPV4 increases in the plasma membrane while decreases in the cytosol (Hu et al., 2019). Our data further suggests that 4αPDD-mediated mineralization could be attenuated by RN1734, suggesting that TRPV4 activation-mediated functions were partly reversed by its inhibition. In several instances, mineralization in response to TRPV4 activation was dose-dependent response. Our data also suggests that the TRPV4-mediated responses and functions seem to be cell-type specific and context-dependent.

Loss-of-TRPV4 gene (*trpv4−/−* animal) results in osteoarthritis and increased bone density compared to wild type (Clark et al., 2010). Although the TRPV4 deficient mice had increased bone density, however, bones are fracture prone and display poor elastic quality. Knock down of TRPV4 also suppresses osteoporosis (Cao et al., 2019). Given that, the TRPV4 deficient mice are viable and are devoid of any major phenotypes, it appears that the loss-of-TRPV4 can be effectively compensated by the other related channels, such as TRPV1 or TRPM8 (Acharya et al., 2021; Chakraborty et al., 2022). Also TRPV1 and TRPV4 double knock out animals show more bone destiny (Nishimura et al., 2020). Moreover, different point mutations in TRPV4 are pathogenic, particularly those having “gain-of-function”, and cause serious bone defects. Moreover, understanding the role of various ion channels during the process of osteogenesis is still in its infancy. Taken together the involvement of different TRP channels in the osteogenesis process is very crucial and more investigation will shed light on this mechanism in future. Detail understanding of TRPV4-mediated osteogenesis requires further study into the underlying signaling pathways, such as possible involvement of BMP, Wnt and Notch pathways. In addition, our findings strongly suggest that mitochondrial parameters are altered due to TRPV4-mediated modulation.

Here we demonstrate that TRPV4 activation affects the intracellular as well as mitochondrial Ca^2+^-levels, mitochondrial energetics and other metabolic parameters. This in general suggests that TRPV4 is involved in the intracellular Ca^2+^-buffering, both at the cytosolic as well as subcellular levels. TRPV4 seems to be involved in regulation of mitochondrial structure–function process also, though the exact mechanism is not clear. We have recently reported that TRPV4 localized in mitochondria and acted as a mitochondrial Ca^2+^-uniporter, and TRPV4 modulation regulated the ER-mitochondria contact sites (Acharya et al., 2022a; Acharya et al., 2022b). In a similar study we have also found that TRPV4 localized in mitochondria of T-cells, and TRPV4 activation increased the mitochondrial Ca^2+^-level (Acharya et al., 2023). Accordingly, we observed that mutational hot-spot region of TRPV4 interacted with Cytochrome C, a mitochondrial protein. This interaction varied in various mutations and was metal ion-dependent (Das et al., 2022). In that context, different point mutants of TRPV4 affect mitochondrial structure, distribution and morphology and functions (such as mitochondrial ROS, Ca^2+^ and ATP) in a variable manner (data not shown). This suggests that TRPV4-mediated channelopathies affect mitochondrial structure-function-metabolism in a “case-by-case” manner and induce a “wide range of abnormalities” (i.e., mild to severe). This also explains the differences in the phenotypic penetration (from very mild change to embryonic lethality) as observed in case of different TRPV4 point mutations (Camacho et al., 2010). Mitochondrial morphology and osteoblast differentiation are interlinked. Recent reports suggest that during the osteogenesis, mitochondria become circular and the aspect ratio increases (Forni et al., 2016; Suh et al., 2023). Osteogenesis is a highly energy-demanding process, and thus mitochondrial energetics and mitochondria-mediated Ca^2+^-homeostasis are directly related to the bone-mineralization process. Therefore, several bone-related disorders originated due to mutation in TRPV4 can be actually due to mitochondrial disorders and/or malfunctions. In that context, our data strongly suggest that TRPV4-mediated channelopathies mostly represent different forms of mitopathy. Taken together, our data suggest endogenous expression of TRPV4 in osteoblasts is relevant for bone-mineralization and these findings have strong bio-medical implications. Future research should describe TRPV4 mutations in relation to mitochondrial function and investigate the mechanism(s) by which TRPV4-mediated skeletal disorders are caused. The significance of mitochondria during osteoblast differentiation and function may be further explored through the detailed characterization of mitochondrial biology. This understanding will have broad implication in many bone-related diseases, such as different forms of bone decay including arthritis.

## Data Availability

The raw data supporting the conclusion of this article will be made available by the authors, without undue reservation.
