# Supplementary material for: TRPV4 regulates osteoblast differentiation and mitochondrial function that are relevant for channelopathy
Source: Front Cell Dev Biol. 2023 Jun 12;11:1066788. doi: 10.3389/fcell.2023.1066788 (PMC10291087; doi:10.3389/fcell.2023.1066788)
Supplement: Supplementary file 1 [file DataSheet1.PDF]

Supplementary information and figures:

TRPV4 regulates osteoblast differentiation and mitochondrial function that are relevant for channelopathy

Tusar Kanta Acharya[1-2]†, Subhashis Pal[3]†, Arijit Ghosh[1-2]†, Shamit Kumar[1-2], Satish Kumar[1], Naibedya Chattopadhyay[3-4], Chandan Goswami[1, 2*]

1. National Institute of Science Education and Research, HBNI, Bhubaneswar, School of Biological Sciences, P.O. Jatni, Khurda 752050, Odisha, India

2. Homi Bhabha National Institute, Training School Complex, Anushakti Nagar, Mumbai 400094, India

3. Division of Endocrinology and Center for Research in Anabolic Skeletal Target in Health and Illness (ASTHI), Central Drug Research Institute (CDRI), Council of Scientific and Industrial Research (CSIR), Lucknow 226031, India.

4. AcSIR, CSIR-Central Drug Research Institute Campus, Lucknow 226031, India.

† Equal contribution

* Correspondence: chandan@niser.ac.in

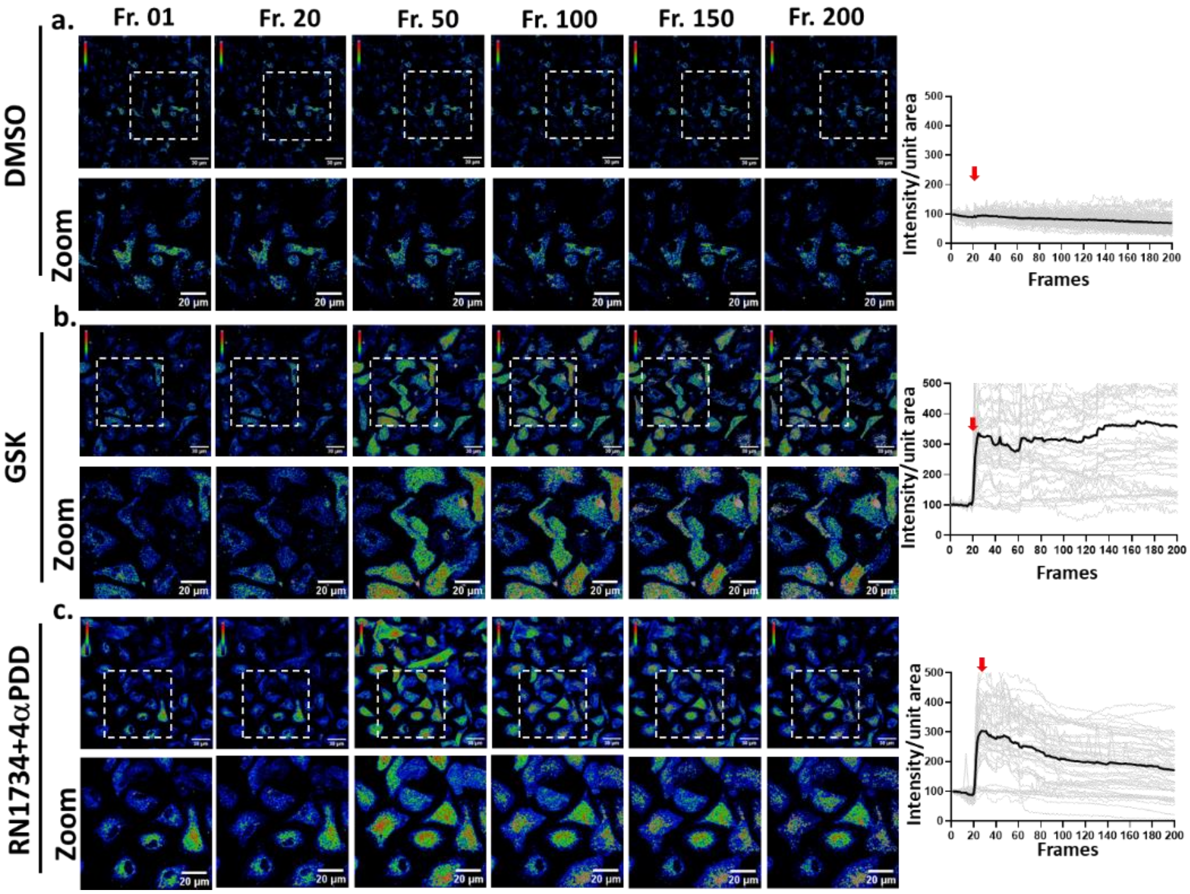

Supplementary Fig. 1. **TRPV4 activation increases cytosolic Ca^{2+} level in mouse bone-marrow derived MSCs**. MSC's were labelled with Fluo-4-AM dye and live cell Ca^{2+} imaging for 200 frames (~3.5mins). **a**. Addition of DMSO does not affect the cytosolic Ca^{2+} levels. **b**. MSC's were treated with GSK1016790A (1µM) at 20th frame to check the effect of instantaneous activation of TRPV4. Activation of TRPV4 by GSK1016790A causes instantaneous Ca^{2+} influx in cells. **c**. The cells were pre-incubated with RN1734 for 1hr, and treated with GSK1016790A at the 20th frame. GSK1016790A treatment increases the cytosolic Ca^{2+} influx but to a lesser extent as compared to only GSK1016790A treated cells. n ≥ 40 cells in each condition.

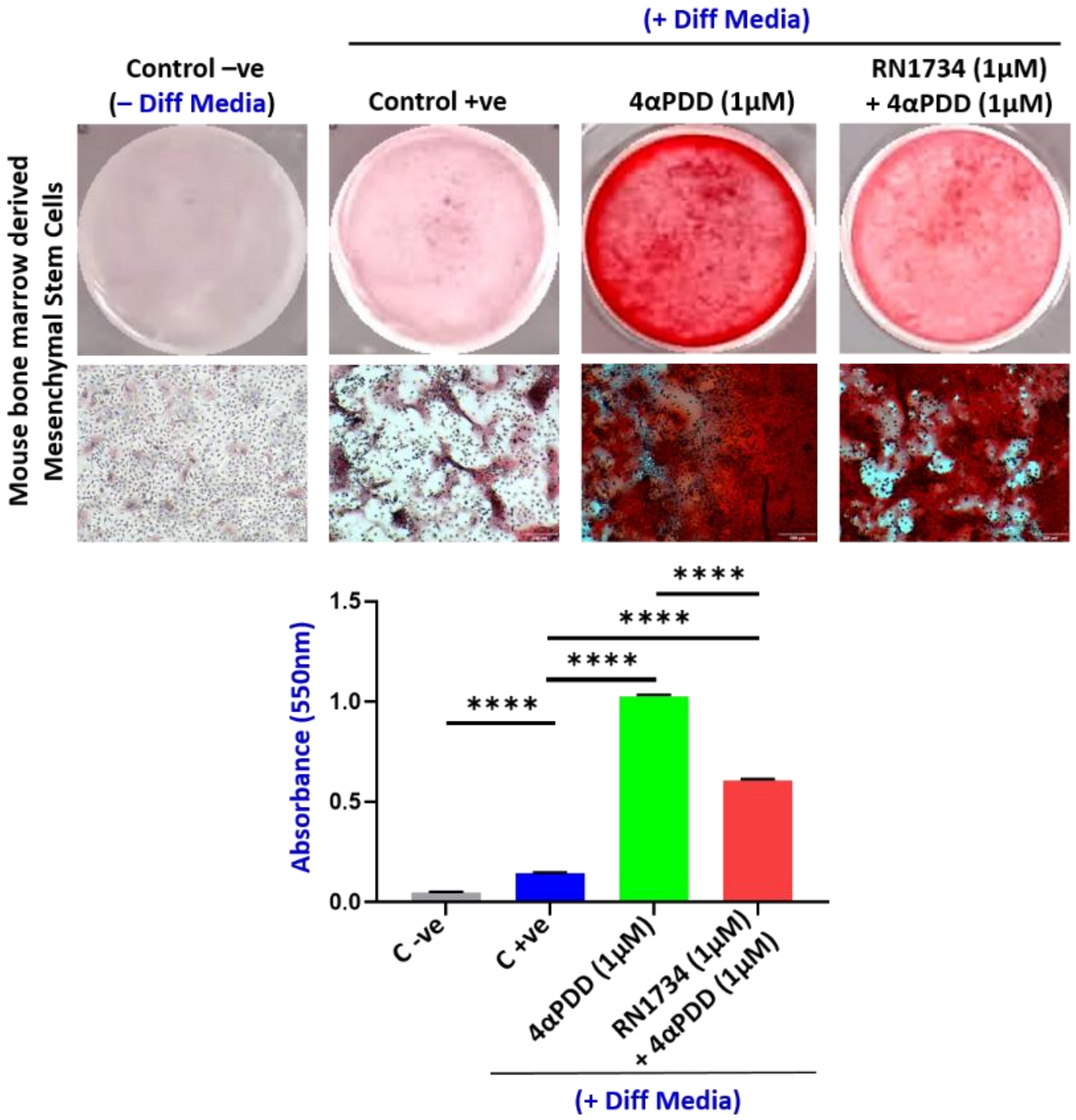

Supplementary Fig. 2. .Mouse bone marrow-derived Mesenchymal Stem Cells (MSCs) were treated with 4αPDD (1µM) only as well as RN1734 (1µM) + 4αPDD (1µM) for 15 days along with osteogenic differentiation media. By performing ARS staining followed by CPC extraction, the extent of mineralization was analyzed. 4αPDD-treated cells have more matrix mineralization as compared to RN1734 + 4αPDD-treated condition. One-way ANOVA. **** = p<0.0001.

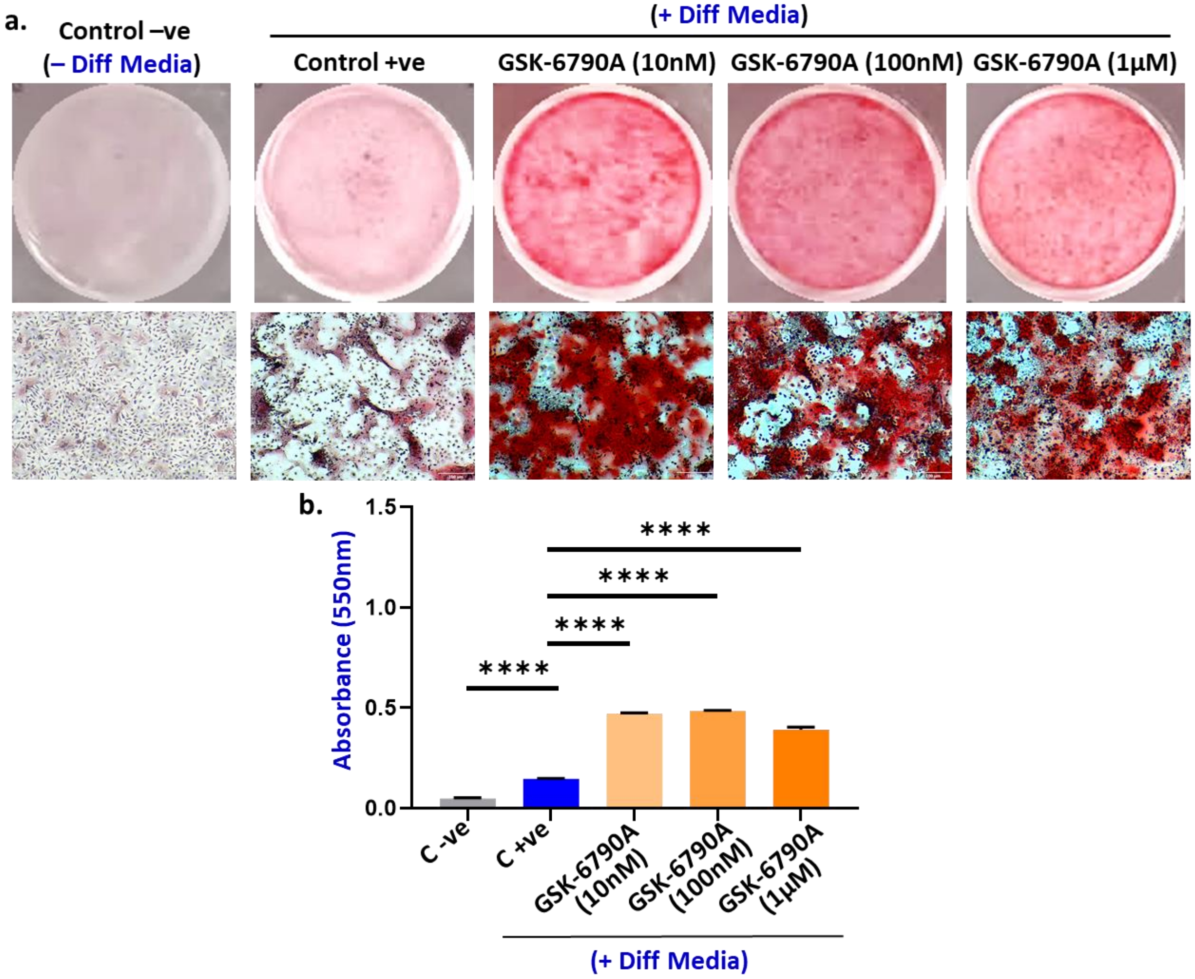

Supplementary Fig. 3. **TRPV4 activation increase osteogenic differentiation in MSCs**. Mouse bone marrow derived mesenchymal stem cells were treated with GSK1016790A to check the level of bone mineralization. **a-b.** ARS staining followed by CPC extraction-based quantification was performed to analyze the extent of osteogenic differentiation. In the presence of GSK1016790A, the level of osteogenic differentiation increases. One-way ANOVA was carried out. **** = $p<0.0001$
